# A Narrative Review on Robotic Surgery as Treatment for Renal Cell Carcinoma with Inferior Vena Cava Thrombus

**DOI:** 10.3390/jcm13051308

**Published:** 2024-02-26

**Authors:** Mihir S. Shah, Kerith R. Wang, Yash B. Shah, Radhika Ragam, Rishabh K. Simhal, Saum Ghodoussipour, Houman Djaladat, James R. Mark, Costas D. Lallas, Thenappan Chandrasekar

**Affiliations:** 1 Department of Urology, Sidney Kimmel Medical College, Thomas Jefferson University, Philadelphia, PA 19107, USA; mihir.shah@jefferson.edu (M.S.S.);; 2 Department of Urology, Oschner Health, New Orleans, LA 70121, USA; 3 Division of Urologic Oncology, Rutgers Cancer Institute of New Jersey and Rutgers Robert Wood Johnson Medical School, New Brunswick, NJ 08901, USA; 4 Institute of Urology, Keck School of Medicine, University of Southern California, Los Angeles, CA 90007, USA; 5 Department of Urology, University of California, Davis, Sacramento, CA 92868, USA

**Keywords:** kidney cancer, IVC level thrombus classification, minimally invasive surgery

## Abstract

Renal cell carcinoma (RCC) is a common diagnosis, of which a notable portion of patients present with an extension into the venous circulation causing an inferior vena cava (IVC) tumor thrombus. Venous extension has significant implications for staging and subsequent treatment planning, with recommendations for more aggressive surgical removal, although associated surgical morbidity and mortality is relatively increased. The methods for surgical removal of RCC with IVC thrombus remain complex, particularly surrounding the use of robot-assisted surgery. Robot assistance for radical nephrectomy in this context is recently emerging. Thrombus level has important implications for surgical technique and prognosis. Other preoperative considerations may include location, laterality, size, and wall invasion. The urology literature on treatment of such tumors is largely limited to case series and institutional studies that describe the feasibility of various surgical options for these complex tumors. Further understanding of the outcomes and patient-specific risk factors would shed increased light on the optimal treatment for such cases. This narrative review provides a thorough overview on the previously reported use of robot-assisted nephrectomy in RCC with IVC thrombus to inform further studies which may optimize outcomes and guide shared decision-making.

## 1. Introduction

Renal cell carcinoma (RCC) is one of the 10 most commonly diagnosed cancers in the United States, affecting approximately 1 in 46 men (2.02%) and 1 in 80 (1.03%) women [1]. Renal vein extension is found in 44% of cases, of which 4–10% have a tumor thrombus within the inferior vena cava (IVC) and 1–16% into the cardiac right atrium; incidence has risen given early detection and increased use of abdominal imaging [2,3,4,5]. Venous extension can drastically alter staging and treatment options.

However, specific approaches for different tumor thrombus levels remain uncertain [6]. The European Association of Urology (EAU) recommends aggressive surgical resection in non-metastatic RCC with IVC thrombus. The current standard of care, in accordance with the National Comprehensive Cancer Network guidelines, is to perform radical nephrectomy (RN) with thrombectomy, although associated surgical mortality is 2–10% [2,7]. Additionally, there is a growing body of literature indicating possible benefits of systemic therapy with delayed surgical intervention [8].

The prognostic implication of the presence and level of IVC thrombus is controversial. Lymph node involvement, metastasis, tumor necrosis, and differentiation have better prognostic value than thrombus levels [5,9,10,11]. However, several studies show that complete resection, regardless of thrombus levels, directly affected survival [11,12,13].

Skinner et al. performed the first open RN with IVC thrombectomy in 1972 [14]. For many years, open surgery was the only option for patients with RCC with IVC extension, especially for cases that required suprahepatic or intrathoracic IVC visualization. The minimally invasive approach to robotic-associated radical nephrectomy with IVC tumor thrombectomy (RARN/IVCTT) is a complex surgery that has significant risk of mortality and morbidity, akin to the open approach, and requires a highly-skilled multidisciplinary team [15]. While the first laparoscopic nephrectomy was performed by Clayman et al. in 1990 [16], Abaza reported the first case series of robotic nephrectomies requiring IVC cross-clamping in 2011 [17]. Since then, several case series of robotic-assisted nephrectomies with cavotomies or IVCTT for different thrombus levels have been described. One of the earliest was described by Ma et al. in 2021, where 20 patients with thrombus levels ranging from 0 to III received RARN/IVCTT [18]. A recent study found significantly improved outcomes with robotic assistance in terms of operative time, blood loss, and volume of blood transfusion [19].

Yet, standardization and guidelines regarding robot-assisted surgery for RCC with IVC thrombus have not been established. This narrative review provides a comprehensive background on robotic surgery for advanced RCC with IVC thrombus to inform continued optimization of RARN/IVCTT and ultimately guide shared decision-making. The paper particularly focuses on details of the surgical techniques of these cases, especially in the robotic approach.

## 2. Methods

We gathered publications for review through PubMed search and citation cross-referencing. Our methodology is summarized in Table 1. We started our investigation by searching for guidelines for renal cell carcinoma management from the American Urological Association, EAU, and National Comprehensive Cancer Network. We continued the search with previous literature reviews on RCC treatment options, institutional and large database studies, and case series. In this manner, we were able to acquire and synthesize a broad sampling of the existing literature. The definitions and outcomes of interest varied between each article.

## 3. Results and Discussion

### 3.1. IVC Level Thrombus Classifications

**Figure 1:**
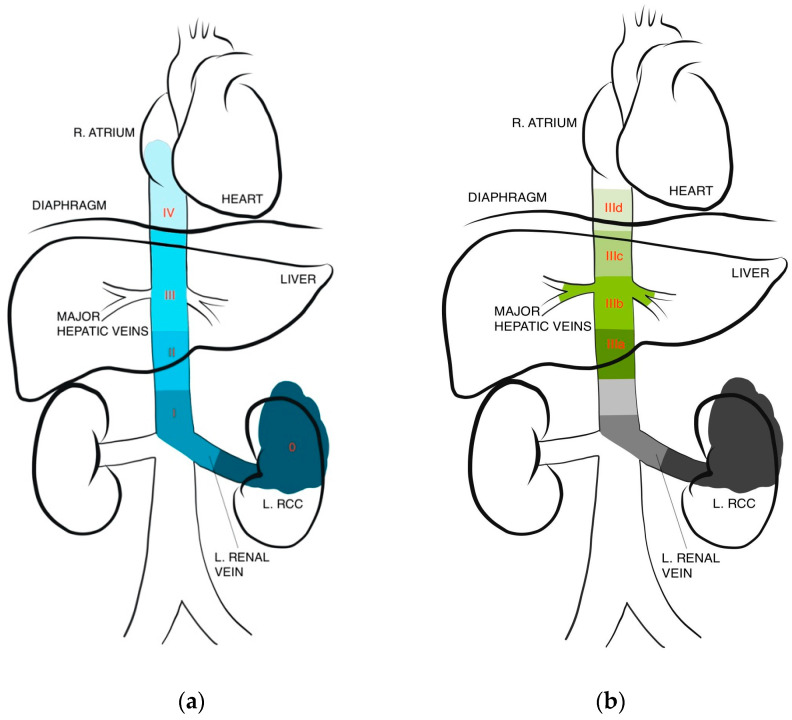
IVC thrombus level classifications. (**a**) Levels I–IV based on Neves and Zincke [21], (**b**) further subclassification of level III based on Ciancio et al. [22].

The extent of tumor thrombus can significantly impact surgical planning and prognosis, making accurate and clinically useful classifications of paramount importance. There are a variety of classifications used in the literature, though their clinical implications have largely been studied only in open surgery. While earlier classifications separated tumor thrombus extension into either two or three levels, typically based on renal vein isolation or infradiaphragmatic versus supradiaphragmatic spread, more recent classifications have increased specificity (Figure 1) [20].

Perhaps the most contemporary system, which is most frequently used in recent literature, is the Mayo classification where RCC IVC thrombi have been classified into four levels [21,23]. Level I involves a thrombus limited to the renal vein or extending <2 cm above, with infrahepatic IVC thrombus denoting level II, thrombus at the level of or above the hepatic veins but below the diaphragm denoting level III, and thrombus entering the right atrium reaching level IV. Notably, studies report that anywhere between 4–16% of patients with thrombi are expected to have extension up to the atrium [5,20]. Ciancio et al. also further subclassify level III thrombi from IIIa to IIId based on ascent per the following: intrahepatic, hepatic, suprahepatic, and supradiaphragmatic. They found the range of dissection and control of the IVC needed within level III alone to be too broad [22]. These modern subclassifications are intended to better facilitate surgical planning while incorporating contemporary considerations such as laparoscopic or robotic assistance. Some authors have similarly proposed subclassifying level II thrombi to facilitate surgical decision-making for those patients.

### 3.2. Surgical Techniques

Radical nephrectomy with tumor thrombectomy remains the gold standard for RCC with IVC tumor thrombus. The principle of management is complete removal of the tumor burden, including tumor thrombus and the involved IVC wall. Studies have shown a greater than two-fold improvement in 5-year survival rates in patients with resection of invaded IVC compared to those without resection [13]. Factors that determine the surgical approach include laterality of the renal tumor given differences in collateral circulation and adjacent structures, as well as the classification, morphology, and extent of tumor thrombus invasion [24]. Table 2 shows a brief overview of the surgical considerations based on the thrombus level.

Despite being the standard of care, RARN/IVCTT remains a challenging operation with significant perioperative morbidity and mortality rates at 2–10% [2]. Traditionally, the open technique has been the standard approach to these challenging and complex cases. However, with significant advancements in minimally invasive surgery, the robotic approach has gained traction over the last two decades. With the robotic approach, there is improved dexterity with arm and wrist maneuvers and enhanced visualization and detail with the surgeon-controlled camera, which enable urologists to perform more precise tissue dissection and suturing [25]. These advances in turn lead to improved retrocaval dissection and minimized IVC manipulation, which decreases the chances of inadvertent thrombus mobilization. Additionally, enhanced visualization and dexterity may both aid in complete caval isolation and control of the infrarenal IVC, suprarenal IVC, contralateral renal vein, and lumbar veins [26].

A meta-analysis by Garg et al. aimed at determining the safety and feasibility of RARN/IVCTT compared to the open approach found that robotic radical nephrectomy with IVC thrombectomy has been shown to have similar oncologic outcomes compared to the open approach, but the minimally invasive approach has improved perioperative outcomes, such as shorter length of stays, lower blood transfusion rates, shorter operating times, and fewer overall postoperative complications [27]. However, there is currently no prospective trial with definitive results or optimal patient selection. There is a paucity of prospective randomized trials that compare outcomes of robotic nephrectomy with IVC thrombectomy to the open approach. Cases are performed by a select number of surgeons every year and with different thrombus levels and varying tumor or patient-related difficulties, and most surgeons perform RARN/IVCTT with either open or robotic approaches but not both. Importantly, the pre-existing data on minimally invasive techniques for RARN/IVCTT are often produced from high-volume centers with abundant expertise [26].

General major steps to RARN/IVCTT include vascular dissection and control in a sequential fashion, cavotomy with excision of tumor thrombus and any bland thrombus, cavotomy repair with heparinization and irrigation, and radical nephrectomy with or without ipsilateral retroperitoneal lymph node dissection.

### 3.3. Right-Sided Tumor

With right-sided cases, the patient may be positioned in the right flank position with subsequent robot docking. They can remain in this position for the entirety of the case, including both the tumor thrombus excision and nephrectomy portions. The right renal artery is dissected and ligated prior to the IVC thrombectomy. Next, inter-aortocaval dissection is performed to gain vascular control, first clamping the infrarenal IVC inferior to any bland thrombus, followed by the left renal vein and infrahepatic IVC. Occlusion or cinching of these major vessels and tributaries is most commonly performed with vessel loop Rummel tourniquets, with some surgeons also using vascular Bulldog clamps [26]. Once hemodynamic stability is confirmed, an L-shaped cavotomy is made along the IVC and to the renal vein so that the thrombus can be dissected from the endothelium. Some reports have also utilized transesophageal echocardiography, particularly for level III thrombi, to assess potential dislodging and subsequent pulmonary embolism [28]. Cavotomy repair is carefully performed to ensure the caval lumen is not significantly narrowed. Vascular flow is then re-established after the tourniquets are removed sequentially as follows: intrahepatic IVC, left renal vein, and then infrarenal IVC. The specimen including the tumor and ipsilateral lymph nodes are secured in a bag and extracted en bloc [29].

### 3.4. Left-Sided Tumor

On the contrary, with the cavectomy-first approach, left-sided cases require positioning in right decubitus for the thrombectomy portion, with repositioning into left flank position and robot re-docking for the nephrectomy portion. Additionally, preoperative left renal artery angioembolization can be performed as the left renal vein will be transected much before robotic control of the left renal artery during radical nephrectomy. Vascular control and IVC tumor thrombus excision with cavotomy repair are performed in a similar fashion as the right-sided thrombus. The left renal vein is stapled, with subsequent clamping of the infrarenal IVC, right renal vein, and intrahepatic IVC [30]. Certainly, right renal vein control requires simultaneous artery control, and oblique infrarenal IVC control may be a preferred alternative. The excised tumor thrombus with or without IVC excision are placed in a bag and removed via the assistant port site. The patient is then repositioned in the left flank position, and the robot is re-docked for completion of the left radical nephrectomy. Alternatively, if preoperative angioembolization is not utilized, then the patient would need to be placed in the left flank position with the robot docked to allow for left renal artery control and ligation followed by redocking in the right flank position to perform the caval thrombectomy. The authors’ preference here is to utilize preoperative angioembolization within 24 h prior to allow for a cava-first approach [29].

### 3.5. Case Reports and Studies for Level I and II

For level I thrombus with minimal extension into the IVC, a cavotomy may not be needed. The thrombus may be milked back into the renal vein or may automatically recede upon visualization of the renal artery and lateral traction of the kidney. Ultimately, this facilitates an adequate renal vein stump for the placement of a clip or the application of a vascular stapler. Conversely, level II cases require IVC manipulation and mobilization, as well as control of the contralateral renal vein, to fully isolate the tumor thrombus [26].

Level I and II thrombi are infrahepatic and often removed via IVC thrombectomy. If the tumor has invaded the vessel wall, then cavectomy may be needed for complete extraction of the thrombus. In a right-sided tumor, the IVC is cross-clamped above and below the tumor thrombus, and the left renal vein is cross clamped as well during the thrombectomy. Similarly, for the left-sided tumor, the contralateral renal vein must be controlled (with or without renal artery control) and cross-clamped in addition to the IVC above and below the tumor [5]. This also warrants consideration of tributaries including corresponding adrenal and lumbar veins [31].

Several series have reported on robotic nephrectomy and IVC thrombectomy [32]. In 2011, Abaza et al. performed the first series on five patients with right-sided tumors [17]. These cases required cross-clamping of the IVC with different techniques, as one patient had aortic stenosis while another patient had two tumor thrombi. Many other studies have also shown that RARN/IVCTT is a feasible option for both left- and right-sided RCC, with promising perioperative data including no or few Clavien grade I-II complications, though 23/30 (76.67%) cavectomy and 12/60 (20%) thrombectomy patients required intraoperative transfusion in one study (Table 3) [33,34,35,36]. The length of stay across seven studies ranged from 1 to 9 days. Two studies reported no complications in 6 out of 6 patients, one of which mentioned adequate pain control without narcotics [17,37]. Most complications experienced were Clavien grade I or II, such as leg edema, acute on chronic renal failure, or blood transfusions. One grade IV complication was reported with a patient experiencing bleeding from tributaries of the IVC with resolution using intraoperative endoscopic suture [35].

### 3.6. Case Reports and Studies for Level III

The challenges and risks of robotic surgery for level III thrombi significantly increase as the liver is often mobilized, and control of the porta hepatis, suprahepatic, and infradiaphragmatic IVC must be obtained for clamping. Some urologists may request assistance from hepatobiliary surgery for liver mobilization [39]. Accordingly, risks of intraoperative blood transfusions and postoperative grade III and IV complications are increased [29,34,40]. Additionally, if the tumor has invaded the IVC wall, a cavectomy may be needed and is typically followed by reconstruction with a graft, although IVC ligation without reconstruction has been demonstrated to yield acceptable near-term outcomes [41,42]. Gill et al. were the first to publish on their experiences with RARN/IVCTT for level III thrombi. A total of 9 patients, 6 with right-sided RCC and 3 with left-sided RCC had operations performed completely robotically with emphasis on “IVC-first, kidney-last”, “minimal-touch”, and “midline-first, lateral-last” techniques [29]. These techniques had been developed to minimize the chance of pulmonary embolism from intraoperative tumor dislodgement, a major complication with high mortality rates that may occur during IVC thrombectomy. 

Right-sided tumors with level III tumor thrombus are approached robotically by first obtaining adequate control of the IVC superior and inferior to the tumor thrombus which is done using a Rummel tourniquet [29]. This dissection requires ligation of all the lumbar veins, short hepatic veins, and adrenal vein as needed to obtain adequate the proximal and distal of the IVC depending on the level of the tumor thrombus. A Fogarty balloon may be used to obtain proximal access above the tumor thrombus [29]. Additionally, the contralateral renal vein as well as ipsilateral renal veins are controlled with a Rummel tourniquet. The renal artery is isolated and then transected using an Endo GIA stapler. Next, the IVC and contralateral renal vein are sequentially clamped. Cavotomy is made and the tumor thrombus is excised en bloc with the renal vein and kidney. The IVC is then repaired robotically using a Gortex suture. The IVC is then sequentially unclamped. A right-sided robotic nephrectomy is then completed. 

Left-sided renal tumors with a level III tumor thrombus are approached in IVC first [27]. This usually necessitates pre-operative angioembolization of the left renal artery, which allows for the surgeon to approach the IVC tumor thrombus first [29]. Dissection of the IVC is the same as described for right-sided tumors. Upon completion of the repair of the cavotomy, the IVC tumor thrombus is resected up to the level of the interaortocaval renal vein using an Endo GIA, and the surgeon then directs their attention to the left nephrectomy portion of the procedure and resects the stapled renal vein stump en bloc with the rest of the left kidney. 

Perioperative outcomes of several case reports and case studies are presented in Table 4.

### 3.7. Case Reports and Studies for Level IV

In the past, level IV IVC thrombectomies were typically performed through a large abdominal incision and median sternotomy or thoracoabdominal incision. The addition of a cardiopulmonary bypass (CPB) with circulatory arrest time, porta hepatis clamping time, and IVC clamping time decrease bleeding but add significant complexity to a procedure with high risk of hemorrhage and need for blood transfusion [44]. The laparoscopic approach has been reported by some, but to our knowledge, the first robotic treatments for a level IV thrombectomy were performed in 2020. Gill et al. performed an RARN/IVCTT in a 74-year-old male with right-sided RCC [45]. A 6 cm mini thoracotomy was performed to facilitate clamping of the aorta, occlusion of the superior vena cava, CPB, and the extraction of the cephalic portion of the thrombus. Wang et al., who previously reported a series of RARN/IVCTTs on level I and II thrombi, also reported a series of RARN/IVCTTs on 6 patients with level IV thrombi in 2020 [35,44]. One of the six cases avoided CPB and was treated like a level III thrombus with the tumor milked out of the atrium into the IVC. There were no intraoperative mortalities, but all required intraoperative blood transfusions. One patient died on postoperative day one in the intensive care unit due to extensive blood loss potentially due to a coagulation disorder. Three patients had Clavien grade II complications, and two had grade IV complications [44]. A hybrid robotic and open approach was reported in 2015 for a left-sided tumor with level IV thrombus. A “minimal touch” technique was first used with the robot during the nephrectomy portion to decrease the risk of tumor thrombus dislodgement. The robot was used for initial dissection and exposure of the kidney and IVC while maintaining hemostasis prior to the anticipated CPB with heparinization and hypothermia. During the robotic portion, the renal artery was ligated first. The kidney was mobilized except for the renal vein, and the IVC was also exposed. Then the patient was repositioned for an open approach, and the cardiothoracic team initiated CPB for the atrial thrombectomy, nephrectomy, and IVC reconstruction steps. While the kidney and renal vein with the attached thrombus were removed en bloc, the cardiothoracic team removed the right atrial tumor. Both the right atrium and the IVC were reconstructed [46]. Additional perioperative data on these cases are shown in Table 5. In general, reports on the challenging removal of level IV thrombi robotically are limited, and this remains largely uncharted territory.

### 3.8. Preoperative Evaluation

Preoperative planning helps determine the success of the RARN/IVCTT, a complicated procedure. Crucial factors to obtain from preoperative imaging include the proximal extent of the thrombus, size of the primary tumor, lymph node status, volume of the thrombus, distance from the hepatic veins, arterialization, and potential caval wall invasion [47,48]. IVC tumor thrombi have the potential to grow rapidly, so it is recommended that imaging be performed or repeated within 1 to 2 weeks of the surgery for precise surgical planning [29]. Gohji et al. found that seven of seven patients with an IVC greater than 40 mm on preoperative computed tomography (CT) had potential vessel wall invasion requiring partial CPB and caval wall repair with a graft. Comparatively, only two of eleven patients with an IVC less than 40 mm on CT required caval wall repair with a graft without CPB [49]. Zini et al. also found the anteroposterior diameter of the IVC to be indicative of vessel wall invasion with a 90% sensitivity when combined with a measurement of the renal vein ostium diameter. This was predictive with an IVC diameter of 18 mm and a renal vein ostium of 14 mm [50].

Of note, there have been studies evaluating the need for cavectomy in the presence of certain patient-specific factors [36]. Using radiographic features, Psutka et al. found that IVC resection was warranted in patients with right-sided tumors, with an IVC diameter over 24 mm, and with complete occlusion of the IVC [51]. Such a model is helpful in preoperative planning and vascular surgery consultation, given the possibility for potential vascular reconstruction.

Magnetic resonance imaging (MRI) has shown better sensitivity than CT for detecting an IVC thrombus, nearing 100% versus 65%, while also reducing radiation and providing multi-dimensional views of the relationships between the tumor and important nearby structures [52,53]. With the improvement of multi-detector CTs that reconstruct thin cuts into multi-planar images, detection of the proximal extent of tumor thrombus, wall invasion, and nodal disease by CT is now on par with MRI [52,54,55,56]. Multi-detector CTs are a reliable alternative for patients with implants or patients who cannot remain still for an MRI. The surgeon should request a three-dimensional reconstruction of the vascular anatomy as part of preoperative imaging to help with surgical planning.

### 3.9. Preoperative Renal Artery Embolization

Currently, there are no guidelines for the types of cases that would benefit from preoperative renal artery embolization (RAE), which are generally performed at the surgeon’s discretion. Most studies comparing the outcomes of patients with or without RAE have been retrospective, thus reporting varying results and conclusions. Generally, angioembolization is performed for patients with a left-sided or large tumor, significant collateral formation, arterialized thrombus, or significant hilar lymphadenopathy [29]. It allows the renal vein, which lies anterior, to be ligated before identification of the renal artery without the increasing risk of blood collection in the kidney or hemorrhage from venous collaterals. It also provides the option of performing the cavectomy or thrombectomy portion of the procedure prior to surgical dissection and ligation of the renal artery.

RAE has been thought to potentially decrease the tumor burden and reduce the cephalad extent of the tumor since the major blood source of the thrombus is the renal artery [23]. This, in turn, decreases intraoperative bleeding and operative time. A retrospective study found that patients chosen to undergo preoperative RAE had significantly larger tumor size at baseline. During the nephrectomy, these patients had less estimated blood loss and transfusion requirements. However, there was no difference in the duration of operation, intensive care unit stay, hospital length of stay, or perioperative complications [57]. Another retrospective study found that patients with preoperative RAE had a 5-year survival rate of 62% and a 10-year survival rate of 47%, which was significantly higher than 35% and 23%, respectively, in the case-matched control group without RAE. This cohort included patients with pT2 and pT3 disease as well as those with lymph node involvement [58]. Some proponents have postulated that the survival benefits of preoperative RAE are due to an immune response with the necrotizing tumor activating natural killer cells, lymphocytes, and macrophages [59,60,61].

The most common complication of RAE is postinfarction syndrome, which manifests as fevers, chills, flank pain, malaise, hematuria, transient hypertension, and hyponatremia due to an immune response to the infarcted kidney [26]. Although mild and self-limited, these symptoms can be reported in about 75% of patients [62]. One large retrospective study found that RAE did not increase survival and actually increased the need for blood transfusions; no other complication rates were affected [63]. Subramanian et al. conducted a prospective study of 225 patients and similarly found that patients who underwent preoperative RAE needed significantly more blood transfusions perioperatively. Additionally, they required longer operation time, exhibited more postoperative complications, and required longer intensive care unit stays, and those with preoperative RAE had five times the odds of perioperative mortality [64].

### 3.10. Multidisciplinary Team

Given the complexity of RARN/IVCTTs and the variety of surgical techniques that draw on several specialties, a multidisciplinary care team and involvement of surgeons with different expertise is often needed. Gayed et al. found that having an experienced team, consisting of the same urologic oncologist, cardiothoracic surgeon, cardiac anesthesiologist, and cardiac scrub team, significantly decreased the rate of overall and major complications [65]. Alternatively, Master et al. did not find a decrease in complications but still found a significant decrease in operation time, intensive care unit admission, and length of stay for patients who underwent RARN/IVCTT with a dedicated team established. All patients were operated on by the same urological oncologist, but the dedicated team included a hepatopancreaticobiliary trained surgical oncologist [66]. A multidisciplinary approach is especially important when considering surgical intervention for level III and IV thrombi.

## 4. Conclusions

This narrative review provides a comprehensive summary of the existing literature on robotic management of RCC with venous extension along with an integration of contemporary single surgeon experiences and case series exploring novel surgical techniques. Overall, our findings suggest that this highly morbid condition remains difficult to treat, and, while open radical nephrectomy with tumor thrombectomy remains the standard treatment, novel approaches have demonstrated success and should continue to be explored. A variety of options can prove useful in difficult surgical scenarios. Such innovative techniques mandate the use of multidisciplinary teams with highly skilled surgeons, and patient selection remains paramount as tumor laterality, thrombus location, and routine surgical contraindications can pose magnified risks in this population. Advanced open and robotic surgery skill, along with a low threshold for potential robot-to-open conversion, can prove beneficial during the learning phase. Overall, this review supports the need for continued research into innovative surgical approaches for RCC with venous extension.

## Figures and Tables

**Table 1:** Search methodology for narrative review.

Items	Specification
Date of search (specified to date, month, and year	18 January 2023–15 February 2023
Databases and other sources searched	Pubmed, Thomas Jefferson University library system
Search terms used (including MeSH and free text search terms and filters) Note: please use an independent supplement table to present detailed search strategy of one database as an example	“Renal cell carcinoma”, “IVC thrombus”, “Thrombus level”, “Robotic surgery”, “Minimally invasive”, “Laparoscopic surgery”, “IVC thrombus level classification”, “Surgical techniques”
Timeframe	To present
Inclusion and exclusion criteria (study type, language restrictions, etc.)	English language, all study types
Selection process (who conducted the selection, whether it was conducted independently, how consensus was obtained, etc.)	KW, YS, RR searched for literature independently; papers were shared and reviewed by all to reach consensus for inclusion
Any additional considerations, if applicable	

**Table 2:** RCC tumor thrombus level classification based on Neves and Zincke and Ciancio et al. [21,22,23].

Level	Description	Surgical Approach
0	Limited to renal vein	Renal vein ligation
I	In the renal vein, <2 cm above renal vein	Tumor can be milked into the renal vein, clamp renal vein
II	>2 cm above renal vein, below hepatic veins	Rummel tourniquet or clamps on, clamping of intrahepatic IVC just below major hepatic veins
III	Above hepatic veins	Requires mobilization of the liver, intraoperative transesophageal electrocardiography, potential clamping of the hepatic veins and cardiopulmonary or venovenous bypass, clamping of suprahepatic IVC
IIIa	Retrohepatic IVC but below hepatic veins	
IIIb	Retrohepatic IVC reaching the hepatic veins, may extend into hepatic veins	
IIIc	Suprahepatic but subdiaphragmatic	
IIId	Supradiaphragmatic	
IV	Into the right atrium	Thoracic and abdominal approach, involves cardiothoracic surgery, cardiopulmonary bypass, and circulatory arrest

**Table 3:** Perioperative results of robotic nephrectomy and IVC thrombectomy for level I–II RCC.

Author/Year	Thrombus Level: N	Side	Approach	Operation Time (min) Mean/Median (Range)	Blood Loss (mL) Mean/Median (Range)	Hospital Stay (Days) Mean/Median (Range)	Complication Rate	Other Findings
Abaza 2011 [17]	I: 5	Right	Left lateral decubitus position at 90°	327 (240–411)	170 (50–400	1.2 (1–2)	0	All patients managed with oral pain control and ketorolac; no IV narcotics No recurrence of disease at a mean 15.4 month follow up
Motoyama 2021 [37]	I: 1	Right	Transperitoneal	211	150	5	0	
Wang 2016 [35]	I: 4 II: 13	Right: 13 Left: 4	Left lateral decubitus position with 70° bump	Right: 131 (100–150) Left: 250 (190–275)	240 (145–320)	5.2 (4–6)	2 out of 17 grade II—postoperative hypoproteinemia with lymphatic leakage grade IV—bleeding from tributaries of IVC	IVC clamping time: 17 min (12–25 min) No recurrence or tumor emboli infringement at IVC at mean 14 month follow up Left RCC: Right-side warm ischemic time 18 min (14–22 min)
Aghazadeh and Goh 2018 [33]	II: 1	Left	Supine Single dock Switch camera ports between thrombectomy and nephrectomy	420	500	5	grade I—acute on chronic renal failure	IVC clamping time: 27 min No recurrence of disease at 12 mos No renal artery embolization or repositioning for a L-sided RCC
Du 2020 [34]	II: 5 III: 6	Left	30–45° dorsal elevated lithotomy	420	1700	3 ICU 6 total	4 Clavien grade II in 2 patients—lower extremity edema, diuretics required	First segmental IVC resection without caval replacement or shunt reconstruction
Shao 2015 [38]	II: 6	Right	Left lateral decubitus position	155 (135–210)	271 (150–510)	9	2 patients with grade I–II complications	IVC clamping time: 16.5 min (13–20 min) No recurrence of disease at a mean 32.5 mo (16–52 mos) follow up
Shi 2020 [36]	II: 90	R: 65 L: 25	Information not available	Thrombectomy: 190 Cavectomy: 268	Thrombectomy: 400 (200–1000) Cavectomy: 1500 (970–2000)		Thrombectomy: 12/60 required intraoperative blood transfusion grade I–II Cavectomy: 23/30 required intraoperative blood transfusion, 2 grade II—bleeding from spleen injury and fistula from intestinal injury, both requiring reoperation	Compared thrombectomies to cavectomies At mean 18 mo (1–75 mos) follow up, thrombectomy: new metastases in 17/60 patients and 6 deaths; cavectomy: new metastases in 19/30 patients and 13 deaths

**Table 4:** Perioperative results of robotic nephrectomy and IVC thrombectomy for level III RCC.

Author/Year	Thrombus Level: N	Side	Approach	Operation Time (min) Mean/Median (Range)	Blood Loss (mL) Mean/Median (Range)	Hospital Stay (Days) Mean/Median (Range)	Complication Rate	Other Findings
Chopra 2017 [40]	II: 13 III: 11	R:17 L: 7	75° lateral decubitus position with table fully flexed R: whole procedure with right side up L: right side up first for thrombectomy, then repositioned left side up for rest of procedure	270 (180–480)	240 (100–7000)	4 (1–22)	5 received intraoperative transfusions 2 grade II—DVT, PE 1 grade IIIa—chylous ascites 1 grade IIIb—subphrenic abscess	“IVC first, kidney last” technique with minimal IVC touch 3 patients had positive lymph nodes At median follow up 16 mo (12–39 mos), all patients were alive; 11 had new-onset metastatic disease; 10 received adjuvant therapy
Gill 2015 [29]	III: 9	R: 6 L: 3	R: Right side up, 60° lateral position L: Right side up first for thrombectomy, then repositioned left side up for nephrectomy and lymphadenopathy	306 (270–378)	493 (200–7000)	6.8 (2–10)	1 grade IIIb—subphrenic abscess 3 required intraoperative transfusion	No evidence of disease or progression at median follow up 7 mo (1–18 mos)
Grosso 2022 [43]	III: 1	Left	Step 1: Right flank Step 2: Supine Step 3: Left flank	600	400	6	None	IVC clamping time: 15 min
Ramirez 2016 [39]	III: 1	Right	Modified left lateral decubitus position with 60° table flexion at the anterior superior iliac spine	353	150	3	None	IVC clamping time: 39 min
Wang 2020 [44]	III: 7 Plus 1 level IV treated like a level III	R: 3 L: 4	30–45° dorsal elevated lithotomy for liver mobilization Repositioned to left lateral decubitus position with 70° bump for thrombectomy	430 (355–550)	1100 (800–2600)	11.3 ± 1.9	1 grade I 1 grade II 1 grade IV 7/8 (87.5%) required transfusion	1 progression of metastases at median follow up 18 mo (12–37 mos) Included one case of urothelial carcinoma on postoperative histology

**Table 5:** Perioperative results of robotic nephrectomy and IVC thrombectomy for level IV RCC.

Author/Year	Thrombus Level: N	Side	Approach	Operation Time (min) Mean/Median (Range)	Blood Loss (mL) Mean/Median (Range)	Hospital Stay (Days) Mean/Median (Range)	Complication Rate	Other Findings
Gill 2020 [45]	IV: 1	Right	6 cm mini thoracotomy Anterograde-retrograde approach for thrombectomy CPB with hypothermic cardiac arrest	Information not available	Information not available	6	Information not available	Follow up 3.5 years later, on immunotherapy for lung metastases
Palma-Zamora 2018 [46]	IV: 1	Left	Hybrid robotic + open procedure Modified left flank position to modified right flank position to supine CPB	224 (robotic portion)	200 (robotic portion)	Information not available	Information not available	“Minimal touch” technique to decrease risk of tumor thrombus dislodgement due to kidney and renal vein manipulation CPB time: 159 min Circulatory arrest time: 25 min Disease free at 18 mo follow up
Wang 2020 [44]	IV: 6 Exclude 1 level IV treated like a level III	Right	30–45° dorsal elevated lithotomy for liver mobilization Repositioned to left lateral decubitus position with 70° bump for thrombectomy CPB 6 cm incision at 5th intercostal for thoracoscopy	510 (338–653)	2800 (1500–6500)	15.4 ± 2.8	1 death POD 1 due to extensive 12,000 mL blood loss 3 grade II 2 grade IV All required transfusion	“Segmented thrombectomy” 1 case underwent “kidney first IVC last” technique CPB time: 72 min (51–87 min) Included one case of urothelial carcinoma on postoperative histology

## Data Availability

No new data was analyzed. Data sharing is not applicable to this article.
